# Sterility of Women

**Published:** 1915-05

**Authors:** 


**Sterility of Women.** R. Schaffer, Zeit. für die Bekámpfung der Geschlectkrankheiten. Of 5,196 married women examined in the polyclinic, 1897-1912, 500 (9.6%) were entirely sterile, i.e., they had not been pregnant even before marriage. 596 (11.5%) had conceived but had aborted or given birth to dead children. Of the 500, 49 were excluded from further statistics by lack of details. Of 451 remaining, entirely sterile, 304 (67.3%) had gonorrhoea. In only 3 cases (0.6%) was the sterility entirely unexplained. Of the 596 who had been pregnant but had not borne live children, 218 were excluded for lack of details. Of the remaining 378, 271 (71%) had gonorrhoea and in only 2 cases was a plausible basis for the sterility lacking.

